# The conserved GTPase LepA contributes mainly to translation initiation in *Escherichia coli*

**DOI:** 10.1093/nar/gku1098

**Published:** 2014-11-06

**Authors:** Rohan Balakrishnan, Kenji Oman, Shinichiro Shoji, Ralf Bundschuh, Kurt Fredrick

**Affiliations:** 1 Ohio State Biochemistry Program, The Ohio State University, Columbus, OH 43210, USA; 2 Center for RNA Biology, The Ohio State University, Columbus, OH 43210, USA; 3 Department of Physics, The Ohio State University, Columbus, OH 43210, USA; 4 Department of Microbiology, The Ohio State University, Columbus, OH 43210, USA; 5 Department of Chemistry and Biochemistry, Division of Hematology, Department of Internal Medicine, The Ohio State University, Columbus, OH 43210, USA

## Abstract

LepA is a paralog of EF-G found in all bacteria. Deletion of *lepA* confers no obvious growth defect in *Escherichia coli*, and the physiological role of LepA remains unknown. Here, we identify nine strains (*ΔdksA*, *ΔmolR1*, *ΔrsgA*, *ΔtatB*, *ΔtonB*, *ΔtolR*, *ΔubiF*, *ΔubiG* or *ΔubiH*) in which *ΔlepA* confers a synthetic growth phenotype. These strains are compromised for gene regulation, ribosome assembly, transport and/or respiration, indicating that LepA contributes to these functions in some way. We also use ribosome profiling to deduce the effects of LepA on translation. We find that loss of LepA alters the average ribosome density (ARD) for hundreds of mRNA coding regions in the cell, substantially reducing ARD in many cases. By contrast, only subtle and codon-specific changes in ribosome distribution along mRNA are seen. These data suggest that LepA contributes mainly to the initiation phase of translation. Consistent with this interpretation, the effect of LepA on ARD is related to the sequence of the Shine–Dalgarno region. Global perturbation of gene expression in the *ΔlepA* mutant likely explains most of its phenotypes.

## INTRODUCTION

Eleven GTPases are universally conserved in bacteria (1,2). These include the well-known translation factors EF-Tu, EF-G and IF2; Ffh, the protein component of the signal recognition particle, and its receptor, FtsY; ribosome assembly factors Era, EngA and YihA; TrmE, a protein involved in tRNA modification; ObgE, a protein implicated in DNA replication and ribosome assembly/regulation and LepA, a translation factor of unknown function.

LepA is a paralog of EF-G encoded upstream of the leader peptidase gene (*lepB*) in *Escherichia coli* (3,4). Despite its universal conservation in bacteria (5), the *lepA* gene can be deleted from the genome without an obvious effect on growth (6). LepA bears considerable similarity to EF-G but localizes largely to the membrane (7,8). The protein domains of LepA are homologous to domains 1 (G domain), 2, 3 and 5 of EF-G (9). LepA lacks regions corresponding to domain 4 and subdomain G′, but has a unique C-terminal domain (CTD). In 2006, Nierhaus *et al.* reported that LepA catalyzes reverse translocation *in vitro* (10). However, more thorough kinetic studies by Cooperman *et al.* have since shown that while LepA can stabilize a pre-translocation (PRE)-like state of the ribosome, LepA preferentially forms this state by binding the PRE complex rather than by catalyzing reverse translocation (11,12). In other words, LepA competes with EF-G for the PRE complex, which may explain how high concentrations of LepA can inhibit translation *in vitro* (10). Structural studies show that, like EF-G, LepA binds the factor-binding site of the ribosome, although it appears to either stabilize or preclude A-site tRNA binding, depending on the experimental conditions (13,14).

The physiological role of LepA has remained elusive. Recently, Nierhaus *et al.* proposed that LepA acts specifically under conditions of stress (e.g. pH 6, 100 mM Mg^2+^) to rescue stalled ribosomes and otherwise is stored in an inactive form at the membrane (8). This idea is based primarily on evidence that *ΔlepA* reduces fitness under these conditions, LepA delocalizes from the membrane under these conditions, and LepA can stimulate poly-Phe synthesis *in vitro* in the presence of high Mg^2+^ concentration. While interesting, these data provide sparse support for the associated model.

In this work, we use ribosome profiling to study the effect of LepA on translation in *E. coli*. We find that, in unstressed and rapidly growing cells, loss of LepA clearly alters translation, mainly the initiation phase.

## MATERIALS AND METHODS

### Biochemical reagents and experiments

Ribosomes, EF-G and mRNAs were purified as described (15–17). LepA was purified multiple times from several overexpression strains as described in the Supplementary Materials and Methods. Purified tRNAs were purchased (Sigma-Aldrich or Chemical Block), and charged and acetylated as described (18). MF-mRNA, identical in sequence to that described previously (10), was transcribed from a linear DNA template using T7 RNA polymerase. This template was generated by extension of annealed oligonucleotides (19), native gel purification and polymerase chain reaction (PCR) amplification. Toeprinting experiments were performed as detailed previously (17), unless otherwise specified. GTP hydrolysis was measured using thin-layer chromatography, as described (18).

### Bacterial strains and plasmids

The *lepA* gene was deleted and replaced with the chloramphenicol (Cm) acetyltransferase (*cat*) gene by the recombineering method (20). Briefly, a DNA fragment was amplified from the template pKD3 using primers #100 (5′-CCGGGCATTAAGGCACAATAATCATACTTTCATATGAATATCCTCCTTA-3′) and #101 (5′-AAACATATTCGCCATGCCAACTCCTAAGGGGTGTAGGCTGGAGCTGCTTC-3′), generating a cassette that contains the *cat* gene between sequences of the *E. coli* genome that normally flank *lepA*. This cassette was moved into BW25113(pKD46) by electroporation, and recombinants were selected on Luria Broth (LB) plates containing 25 μg/ml Cm. This *ΔlepA::cat* mutation was then moved by P1 transduction into BW25113 (21) to generate KLF2604, into the Hfr strain PK191 (Coli Genetic Stock Center, Yale University) to generate KLF2574, and into the Hfr strain KL16 (CGSC) to generate KLF2573. KLF2604 was used in the ribosome profiling experiments, and KLF2573 and KLF2574 were used as donors in the synthetic genetic screen.

To make pRB34, the H81A mutation was introduced into pLEPA (22) using QuikChange^TM^ mutagenesis (Stratagene) and primers 5′-ATCGACACCCCGGGCGCCGTAGACTTCTCCTATG-3′ and 5′-CATAGGAGAAGTCTACGGCGCCCGGGGTGTCGAT-3′. The In-Fusion^TM^ (Clontech) method was used to generate pRB35. A DNA fragment corresponding to pLEPA without codons 487–594 of *lepA* was amplified using primers 5′-TAATACGTCTACACGTACCATGTCGGACGCCTGGAA-3′ and 5′-CGTGTAGACGTATTAGGCAAAGACAACAAATAA-3′. This product was then circularized as recommended by the supplier (Clontech) and transformed into the cloning strain C2992. Plasmid pRB35 was purified from the transformed cells and confirmed by sequencing.

### Growth rate measurements

Cells were grown in LB media at 37°C. Growth was monitored by measuring the turbidity of the culture (OD_600_) as a function of time. Data were fit to the equation OD_600_ = *a* · 2*^t/d^*, where *t* represents time, *d* represents the doubling (or generation) time and *a* represents the OD_600_ at time *t* = 0.

### Western and northern analyses

Total lysate from mid-log phase cells were resolved using 8% sodium dodecyl sulphate-polyacrylamide gel electrophoresis (PAGE), and proteins were transferred to nitrocellulose membranes (Hybond-C Extra, GE Healthcare). Polyclonal antibodies raised against purified *E. coli* LepA (Prosci Inc.) were used to probe the membranes, and detection was carried out with the secondary antibody ECL system (GE Healthcare).

Total aminoacyl-tRNA was extracted under acidic conditions and subjected to acid gel electrophoresis and northern analysis, using the tRNA^Gly3^-specific oligonucleotide 5′-[^32^P′-CTCGCGACCCCGACCTTGGCAAG-3′ as described (23).

### Sample preparation for ribosome profiling analysis

Cells of the wild-type (WT) strain [BW25113 containing the empty vector pWSK29 (24)], the *ΔlepA* mutant (M) strain [KLF2604(pWSK29)] and the complemented (C) strain [KLF2604(pLEPA)] were grown in LB media at 37°C to mid-log phase (OD_600_ ≈ 0.5), chilled by pouring over crushed ice and harvested by centrifugation. Pellets were resuspended in 500 μl lysis buffer [10 mM Tris-HCl (pH 8), 10 mM MgCl_2_, 60 mM NH_4_Cl, 1 mg/ml lysozyme, 100 U/ml RNase-free DNase I (Roche)] and 400 U/ml Superase inhibitor (Ambion) and lysed in three freeze/thaw cycles. Clarified lysates were loaded onto 10–40% sucrose gradients in TMN buffer (10 mM Tris-HCl pH 8.0, 10 mM MgCl_2_, 60 mM NH_4_Cl, 2 mM DTT) and spun in an SW-41 rotor (Beckman) for 2.5 h at 35000 revolutions per minute. Gradients were fractionated using a syringe-pump system (Brandel) with in-line ultraviolet absorbance detector (UA-6, ISCO).

To prepare ribosome-footprint libraries, lysates were treated with 3000 U/ml micrococcal nuclease (Roche) in the presence of 5 mM CaCl_2_ for 1 h at room temperature prior to sucrose gradient sedimentation. Monosomes were collected, RNA was extracted using phenol and CHCl_3_ and RNA fragments in the 20–30 size range were gel purified and used to generate cDNA libraries as described (25). To prepare total RNA libraries, RNA was extracted from the lysates using TRIzol^®^ (Life Technologies), fragmented by alkaline hydrolysis, fragments in the 30–40 size range were gel purified and cDNA libraries were made as described (25).

### Sequencing

Barcoded libraries were pooled into two lanes of an Illumina HiSeq 2500 Sequencer, single-end sequenced with a read length of 50 nucleotides (nts) and demultiplexed. For libraries resulting in fewer than 15 million reads, cDNA for the corresponding samples was re-sequenced, and reads obtained were combined with the original reads. Data are available at the NCBI Sequence Read Archive (study accession number SRP048921).

### Adapter trimming and read quality control

In order to eliminate low quality reads, any nt with phred quality score below 20 (99% base call accuracy) was converted to an ‘N’ and any trailing ‘N's were removed. 3′ adapters were identified and removed if at least 5 nts of the 3′ adapter sequence matched the sequenced read from a given nt to the end of the read and there were less than 20% mismatches between the candidate read region and the adapter sequence (doubly penalizing mismatches for any nt if the read did not show an ‘N’ at that location). Finally, post-adapter-removal, any reads shorter than 20 nts or containing more than 5 ‘N's were discarded.

### Read preprocessing and alignment

The remaining reads were aligned to the *E. coli* genome (NCBI Reference Sequence: NC_000913.2) using Bowtie2 v 2.0.0-beta5 (26), using default parameters. This provided an average of 6.9% unaligned, 17.5% uniquely mapped and 75.6% multiply-mapped reads per total-RNA library, while ribosome-footprint libraries had an average of 18.5%, 36.8% and 44.7%, respectively. Based on bowtie2's default settings, for multiply-mapped reads, one location chosen at random from the possible mapping sites was used for downstream analysis. The sam files generated by bowtie2 were then converted to the bam format, sorted and indexed for easy read access using samtools version 0.1.18 (27).

### Computational removal of rRNA reads

Prior to further analyses (unless otherwise stated), the rRNA reads were computationally identified and removed. We downloaded genomic locations for 5S rRNA from the UCSC Genome Browser's Rfam track (28). We also downloaded the sequences of the 5S, 16S and 23S rRNAs from http://ecoliwiki.net/colipedia/index.php/Category:rRNA and used the Basic Local Alignment Search Tool (29) to identify their genomic locations, expanding overlapping rRNA regions to the maximum range. We then removed all unmapped reads and all reads that overlapped any of the annotated rRNA sites. After this removal, 29.8% of the original reads were left in the total RNA samples (55.8% of which were, on average, annotated as uniquely mapping), and 38.7% of ribosome footprint reads remained, with 95% of them being uniquely mapped.

### Assignment of read location

For downstream analysis, each read needed to be associated with a single genomic location. The midpoint of reads was used for total RNA libraries. Ribosome footprints were mapped to the predicted center of the P site of the ribosome. To identify this P site, we created a histogram of 3′-end positions of ribosome-footprint reads (shown to vary in distance from the P site less than the 5′ ends (30)) as a function of the distance from the 3′-end of their containing genes, normalizing each read by the total RNA coverage over gene length for that gene in the corresponding sample type (WT, M, C), ensuring all genes contribute equally in ribosome location placement (see Supplementary Figure S1). This histogram shows a peak 10 nts downstream from the stop codon, which we associate with ribosomes undergoing termination, with the stop codon in the A site (31,32). Thus, we identified the center of the P site to be 14 nts prior to the 3′ ends of ribosome-footprint reads. Histograms of genomic locations (+/− strand coverages combined) of all reads were normalized by the total number of reads after quality control and ribosomal RNA removal to provide coverage in reads per million, combined across replicates for each condition and stored as bed files for import into the UCSC genome browser (28). Strandedness of reads was preserved for all other downstream analysis, unless otherwise stated.

### Statistical tests

All statistical tests were performed using the R statistical programming package version 3.1.0 (R Core Team, 2014).

### Read reproducibility (replicate versus replicate)

To validate reproducibility across replicates, raw read coverages across the genome (with +/− strand coverages combined) were compared from replicate to replicate across WT, mutant (M) and complemented (C) samples for both total RNA and ribosome footprints. The Pearson's correlation coefficient was determined for each of the replicate comparisons (Supplementary Figure S2).

### High coverage genes and overall normalization

To ensure adequate signal-to-noise ratios and minimize the effect of multiple testing, we determined a list of high coverage genes by partitioning reads into genes based on genomic location, as annotated by the *E. coli* K12's Genbank RefSeq gene annotation file (downloaded from the UCSC Microbial Genome Browser on 15 April 2013; (28)). For each of the two types of libraries (total RNA, ribosome footprint), total coverage (all WT, M, C samples) for each gene was determined and genes were then sorted from highest to lowest coverage level. Genes in the top 1/2 of the two lists were compared, and those in the intersection were designated high coverage genes (1872 genes). To account for overall sequencing efficiency differences between libraries, all read coverages for further analyses at any given location were normalized by the total number of coding-region reads for each sample (utilizing all gene annotations).

### Gene by gene overall coverages

Normalized reads were accounted into the different high coverage genes for each library. Average ribosome density (ARD) was determined by taking ribosome footprint coverages per gene and dividing by the coverage of the corresponding replicate of total RNA. To test for significance in ARD fold changes across experimental conditions (WT versus M and M versus C), we took the base 2 logarithm of all gene coverages, and performed a Student's *t*-test, corrected for multiple testing utilizing the Benjamini–Hochberg correction (33).

### Ribosome footprint versus total RNA correlations

Gene-by-gene correlations were performed comparing ribosome footprints coverages $x_i$ with total RNA coverages $y_i$ for the high coverage genes. A least square line of best fit was fitted in fold change (log–log) space, shifting all values up by the minimum non-zero gene coverage in each sample, constraining the *y*-intercept to be zero in the non-log, normalized coverage space (only allowing the slope to be fit). A measure of spread, $d^2$, was calculated in this log–log space, where:

\begin{equation*} d^2 = \frac{1}{n}\sum\nolimits_{i = 1}^n {(\tilde y_\iota - \tilde x_i - \tilde A)^2 } \end{equation*}

and $n = 1872$ (the number of high coverage genes being used in the comparison), $\tilde y_\iota = \ln (y_i + y_{\min } )$ (the logarithm of the shifted total RNA coverage for one gene), $\tilde x_i = \ln (x_i + x_{\min } )$ (the logarithm of the shifted ribosome footprint coverage for that gene) and $\tilde A = \ln (A)\,(A = {\rm the}\,{\rm fitted}\,{\rm slope})$. Student's *t*-test was performed, comparing the differences in spread between WT versus M and M versus C samples.

### Read count comparisons

Read counts from the respective samples were compared for total RNA libraries, comparing total mRNA, rRNA, tRNA and other stable small RNAs to total aligned reads. Significances of LepA effects were tested using Student's *t*-test.

### Translation initiation region (TIR) sequence analysis of LepA-affected genes

We determined purine and pyrimidine frequencies for each position of the TIR for genes that exhibit altered ARD due to loss of LepA (i.e. significant change in both the WT versus M and C versus M comparisons; 283 genes where WT, C < M and 237 genes where WT, C > M). The nt sequence, aligned with respect to the start codon, was obtained for these genes, as well as all high coverage genes (to be used as a background), utilizing the gene annotations and the *E. coli* genome mentioned previously. Genes *infC* and *pcnB* were left out of the analysis, as they have the non-standard start codon AUU. The percentage of purines and pyrimidines was then determined for each list, in a location-dependent manner, and finally, the respective percentages from the LepA-affected genes were divided by the percentages for all high coverage genes to yield an enrichment/suppression of purine and pyrimidine prevalence compared to background levels. Any locations where the LepA-affected and the high-coverage genes had a percentage for purine or pyrimidine prevalence that were both zero were re-labeled to have an enrichment of 1. A two-tailed binomial test was performed for the pyrimidine frequencies for 8–11 nts prior to the start codon.

### Metagene analysis of 5′ ends of genes

Ribosome density (RD) was determined for each gene at every position in the gene by determining:

{\fontsize{7}{}{\fontsize{7}{11}\selectfont\begin{eqnarray*} &&RD = \nonumber \\ &&\frac{{Normalized\,Ribosome\,Footprint\,Coverage}}{{(Normalized\,total\,RNA\,Full\,Gene\,Coverage)/(Gene\,Length)}} \end{eqnarray*}}}

These RDs were then aligned to the 5′ end of each gene and averaged over all high coverage genes.

### Gene windows analysis

Relative ribosome occupancies of high coverage genes were determined for ribosome footprints, analogous to RD, by:

{\fontsize{7}{}{\fontsize{7}{11}\selectfont\begin{eqnarray*} &&c_i = \nonumber \\ &&\frac{{Normalized\,Ribosome\,Footprint\,Coverage}}{{(Total\,Normalized\,Ribosome\,Footprint\,Gene\,Coverage)/(Length\,of\,Gene)}} \end{eqnarray*}}}

where *c_i_* is the relative ribosome occupancy at a given nt, *i*, in the gene, normalized for gene-by-gene coverages (to treat all windows across all genes with equal weight, for downstream analysis). A local coverage comparison across sample types was performed by taking 10 nt windows, shifted by 5 nts at a time, scanning over each gene, comparing the window coverages. Window coverage, per window, is the sum over *c_i_*'s over every nt in the window. Only windows which showed a complemented average window coverage (averaged across replicates per WT, M and C strains) were tested for the significance of the coverage differences (WT versus M and M versus C) utilizing a Benjamini–Hochberg (33) corrected Student's *t*-test.

For the 56 windows deemed significant (*q*-value ≤ 0.05) for both WT versus M and M versus C comparisons, consecutive and overlapping windows were combined, then the local coverages were visually examined. Of the 38 regions examined in genes with enhanced coverages in the mutant (WT, C < M), we identified 25 (treating the largely identical genes *tufA* and *tufB* as a single instance) to contain a single, clear, complemented peak, and examined the peak-aligned codon frequencies across the windows, normalized to background levels across the prevalence of codons in all high coverage genes. A two-tailed binomial test was performed for GGU, which appeared in greater abundance compared to all other codons at the A site of the peaks. Although the 12 regions with reduced coverage in the mutant (WT, C > M) were also examined in a similar manner, no clear complemented peaks were observed.

## RESULTS

### LepA exhibits ribosome-dependent GTPase activity *in vitro* but fails to promote reverse translocation

LepA was purified multiple times from three overexpression strains, including one from the Nierhaus laboratory (see Supplementary Materials and Methods). Reverse translocase activity was tested in various complexes and under various conditions, including those employed in Qin *et al.* (10). We found that LepA catalyzes ribosome-dependent GTP hydrolysis but fails to promote reverse translocation (Supplementary Figures S3–S6). Neither the amplitude nor the rate of spontaneous reverse translocation was increased in the presence of LepA and GTP. These data are consistent with results from the Cooperman group (11,12) and call into question the claim that LepA can catalyze reverse codon–anticodon movement in the ribosome (10).

In one of our initial purifications, we used the cloning strain C2992 and neglected to include protease inhibitors, resulting in the recovery of a truncated version of LepA (LepA-trunc) that lacks 50–60 residues (∼6 kDa) of the C-terminus. A previous crystallographic study of LepA revealed that the last 44 residues of the protein are disordered in the crystal and presumably flexible (9), thus it is not surprising that the C-terminus of LepA is susceptible to proteolysis. Interestingly, LepA-trunc catalyzes forward translocation, albeit with low efficiency (*k*_cat_/*K*_M_ = 0.11 min^−1^ μM^−1^) (Supplementary Figure S6). The rate of translocation in the presence of LepA-trunc is similar to that seen for EF-GΔ4 (0.11 versus 0.48 min^−1^ at 1 μM factor) (34), a variant of EF-G predicted to resemble LepA-trunc in structure.

### Synthetic phenotypes conferred by *ΔlepA*

To gain clues about the physiological role of LepA, we screened for synthetic phenotypes of *ΔlepA* in *E. coli*, using a method described previously (35). The null allele *ΔlepA::cat* was moved by conjugation into each strain of the Keio collection, an ordered set of strains in which each non-essential gene is deleted and replaced with a kanamycin (Kn)-resistance gene (21). Donor and recipient cells were combined (co-spotted) on solid rich media in a 96-spot array, incubated for 10–12 h to allow mating and transferred using a 96-pin replicator onto ‘intermediate selection’ plates containing Kn. After growth for 8–10 h, cells were transferred onto selective plates containing both Cm and Kn. Resulting colonies were visually screened for those exhibiting altered growth relative to the parental strains. Little or no growth was seen for the ‘self-mating’ cross of *ΔlepA::cat* × *ΔlepA::kan*, consistent with a low frequency of chromosomal duplication events under these conditions (35).

We used two different Hfr donor strains—one with the F plasmid integrated near 44 min and oriented to transfer *ΔlepA::cat* in a clockwise manner, and another with F integrated near 65 min and oriented to transfer *ΔlepA::cat* in a counterclockwise manner. Several crosses resulted in smaller or larger colonies compared to the parental strains, regardless of which Hfr donor strain was employed, indicating potential genetic interactions (negative or positive). Reduced growth was seen for crosses involving deletions of *dksA*, *molR1*, *rsgA*, *tatB*, *tolR*, *tonB*, *rseA*, *rseB*, *rseC* and *recO*. Because the latter four genes are closely linked to *lepA*, we suspected that these were ‘false positives’, due to the predicted low probability of generating the double-mutant recombinants. Indeed, when these crosses were further analyzed individually, frequencies of colony formation were about 300-fold lower than crosses involving unlinked markers, and the resulting transconjugants exhibited no obvious growth defects relative to the parent strains. Three crosses of the screen, involving deletions of *ubiF*, *ubiG* and *ubiH*, showed larger colonies on the Cm Kn plates, raising the possibility that the growth phenotypes caused by these mutations were being suppressed by *ΔlepA*. However, subsequent PCR analysis of these selected recombinants indicated the presence of the WT gene (e.g. *ubiF^+^*) in addition to the *kan*-marked null allele (e.g. *ΔubiF::kan*). Thus, something more complex was occurring in these crosses, presumably involving regional duplications of each *ubi* locus.

To clarify which of the candidate genes exhibited negative or positive genetic interactions with *lepA*, we used P1 transduction to construct each double mutant *de novo*. Double mutants harboring *ΔlepA* and either *ΔdksA*, *ΔmolR1*, *ΔrsgA*, *ΔtatB*, *ΔtonB*, *ΔtolR*, *ΔubiF*, *ΔubiG* or *ΔubiH* showed a reduced growth rate, indicating negative interactions (Table 1). In all cases, the reduced growth rate was at least partially complemented by plasmid pLEPA, which carries *lepA* downstream from its native promoter (22). These mutations cause defects in gene regulation (*dksA*, *molR1*) (36,37), ribosome biogenesis (*rsgA*) (38), respiration (*ubiF*, *ubiG*, *ubiH*) (39,40) and/or transport (*tatB*, *tonB*, *tolR*) (41–43), suggesting that LepA contributes to these processes, directly or indirectly.

**Table 1.** Strains in which *ΔlepA* confers a synthetic growth defect

Genetic background^a^	Genome position^b^	Gene function	Doubling time (min)				
			*lepA+*	*ΔlepA*	*ΔlepA* (pLEPA)^c^	*ΔlepA* (pRB35)^d^	*ΔlepA* (pRB34)^e^
Wild-type	NA	NA	24 ± 2	26 ± 1	26 ± 1	ND	ND
*dksA*	3.5	Stringent response, transcriptional regulator	33 ± 1	52 ± 3	34 ± 2	ND	ND
*molR*	47.3	Molybdenum utilization, regulatory protein	22 ± 1	33 ± 2	24 ± 2	ND	ND
*rsgA*	94.6	Ribosome biogenesis, GTPase	26 ± 1	31 ± 1	26 ± 1	ND	ND
*tatB*	86.7	Component of twin-arginine translocase (TAT)	29 ± 1	44 ± 2	35 ± 2	ND	ND
*tonB*	28.2	Component of TonB-Exb system, PMF-dependent transporter	25 ± 1	35 ± 2	30 ± 1	ND	ND
*tolR*	16.7	Component of Tol-Pal system, PMF-dependent transporter	27 ± 1	31 ± 2	28 ± 2	ND	ND
*ubiF*	15.0	Ubiquinone biosynthesis	37 ± 2	58 ± 2	43 ± 2	55 ± 3	57 ± 2
*ubiG*	50.4	Ubiquinone biosynthesis	39 ± 1	64 ± 2	43 ± 2	60 ± 3	61 ± 4
*ubiH*	65.8	Ubiquinone biosynthesis	34 ± 1	42 ± 1	31 ± 1	ND	ND

Reported values represent the mean ± SEM for ≥3 independent experiments. NA, not applicable; ND, not determined.

^a^Single-gene deletions of the Keio collection, each marked with a Kn-resistance cassette. Wild-type, the parental strain BW25113 [*F-*, *λ^−^*, *Δ(araD-araB)567*, *ΔlacZ4787::rrnB-3*, *Δ(rhaD-rhaB)568*, *rph-1*, *hsdR514*].

^b^In units of minutes (or centisomes).

^c^Plasmid pLEPA contains the wild-type *lepA* gene downstream from its native promoter.

^d^Plasmid pRB35 expresses a variant of LepA lacking the CTD (Δ487–594).

^e^Plasmid pRB34 expresses a variant of LepA with H81A.

Among the sickest strains were those in which *ΔlepA* was combined with *ΔubiF*, *ΔubiG* or *ΔubiH*, even though the original screen predicted positive genetic interactions in these cases. We suspect that the original Hfr-mediated crosses involving *ΔubiF*, *ΔubiG* and *ΔubiH* allowed for chromosomal duplication events, resulting in merodiploid recombinants that outgrew the parental *Δubi* strains. While the basis of this effect remains unclear, mutations in these *ubi* genes have been shown to cause partial resistance to aminoglycosides, due to defects in uptake (44,45), which may have altered the kinetics of Kn selection in the Hfr-mediated crosses and thereby enriched for merodiploid recombinants.

### LepA variants lacking the active-site histidine or unique CTD fail to complement the synthetic phenotypes

We took advantage of the synthetic phenotypes to test the functional importance of the CTD and GTPase activity of LepA. Derivatives of pLEPA were constructed, one (pRB35) to express LepA lacking its CTD (Δ487–594) and the other (pRB34) to express LepA carrying mutation H81A. Histidine 81 is predicted to be critical for GTPase activity, as analogous substitutions of EF-Tu and EF-G cause specific loss of GTPase activity (46,47). When transformed into *ΔubiF* *ΔlepA* and *ΔubiG ΔlepA* backgrounds, each plasmid failed to complement the synthetic phenotypes of *ΔlepA* (Table 1). Western analysis showed that the level of mutant LepA protein produced in cells harboring pRB34 or pRB35 is comparable to the level of LepA seen in cells harboring pLEPA, slightly higher than that in WT cells (Supplementary Figure S7). These data provide evidence that both the CTD and the GTPase activity of LepA are critical for its function *in vivo*.

### Many mRNA coding regions exhibit reduced ARD in the absence of LepA

To investigate the role of LepA in translation, we analyzed the WT, *ΔlepA* mutant (M) and *ΔlepA*(pLEPA) complemented (C) strains, using the ribosome profiling technique (25,48). Cells were grown in LB media at 37°C to mid-log phase, rapidly chilled to inhibit translation elongation and lysed (49). Polysomes in the lysates were digested with micrococcal nuclease, the resulting 70S ribosome complexes were isolated from sucrose gradients and the protected mRNA fragments (ribosome footprints) were recovered after phenol extraction using PAGE. In parallel, total RNA was extracted from cells grown under identical conditions, randomly fragmented via base hydrolysis and fragments of a similar size range were PAGE purified. From the ribosome footprint and total RNA samples, cDNA libraries were prepared and analyzed by high-throughput sequencing as described (25). Three independent experiments were performed for each of the strains, resulting in 18 cDNA libraries. Per strain, 45–69 million ribosome-footprint reads and 40–65 million total-RNA reads were aligned to the *E. coli* K12 MG1655 genome, allowing the effects of LepA on global gene expression to be deduced. As in analogous studies, the data obtained were highly reproducible among the biological replicates (Supplementary Figure S2).

**Figure 1.**
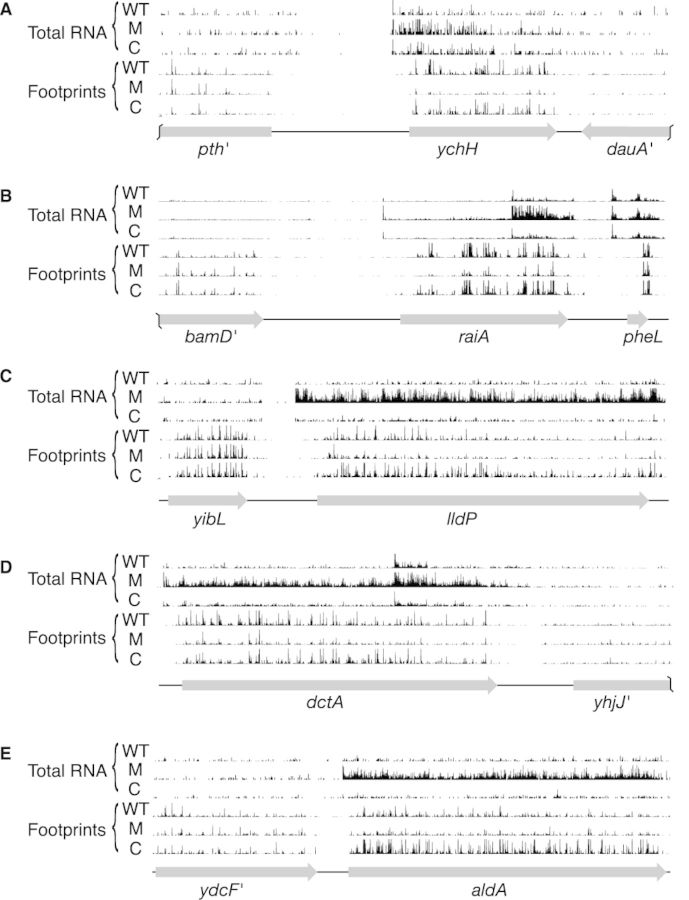
Examples of genes that exhibit decreased ARD in the absence of LepA. Total-RNA and ribosome-footprint read counts for WT, mutant (M) and complemented (C) strains are shown mapped back to the genome in the vicinity of *ychH* (A), *raiA* (B), *lldP* (C), *dctA* (D) and *aldA* (E). Ribosome-footprint reads are mapped to the genomic position corresponding to the predicted central nt of the P codon, and total-RNA reads are mapped to the center of the read fragment. Read counts are normalized with respect to total number of reads (after quality control and rRNA read removal), making the histograms of analogous data tracks directly comparable in each panel.

**Figure 2.**
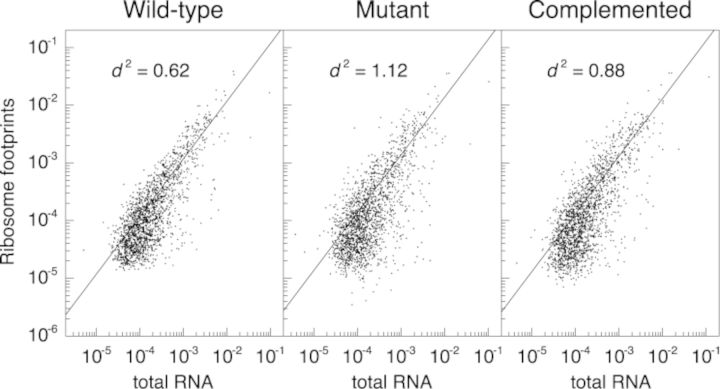
Gene expression is globally perturbed in the absence of LepA. Normalized gene-by-gene coverages are compared between ribosome footprints and total RNA in WT, mutant and complemented strains (as indicated). A measure of spread, $d^2$, was calculated for each of the comparisons, yielding 0.62 ± 0.02, 1.12 ± 0.02 and 0.88 ± 0.03 for the WT, M and C samples, respectively. Student's *t*-tests on the values of $d^2$ in all three replicates yield *P* = 1.6 × 10^−5^ for the WT versus M, and *P* = 1.2 × 10^−3^ for the M versus C comparison, showing significant perturbation of translation efficiencies (i.e. changes in ARD values) due to loss of LepA.

Loss of LepA substantially reduced the average ribosome density (ARD) for many coding regions (Supplementary Tables S1–S3). Of 1872 genes analyzed (the most-highly expressed half), 520 had significantly altered ARD due to loss of LepA (i.e. both WT versus M and C versus M comparisons yield Benjamini–Hochberg corrected *q*-values of ≤0.05 (33)). Twenty-six genes showed reduced ribosome densities of 10-fold or greater in the mutant strain (Table 2), whereas only one gene (*flu*, encoding Antigen 43 autotransporter) showed increased ARD of this magnitude. Displayed in Figure 1 are profiling data for several genes with substantially reduced ARD in the absence of LepA. In nearly all such cases, the data indicate a decrease in protein production, as indicated by fewer ribosome-protected fragments, as well as an increase in mRNA level. We suspect that the high mRNA level is due to transcriptional regulation in response to the primary translational defect conferred by *ΔlepA*. The degree to which *ΔlepA* globally perturbs gene expression (i.e. the collective processes of transcription and translation) can be seen in plots comparing gene-by-gene coverage of ribosome footprints versus total RNA (Figure 2), which give spread coefficients of 0.62 ± 0.02, 1.12 ± 0.02 and 0.88 ± 0.03, in the WT, M and C strains, respectively.

**Table 2.** Coding regions that exhibit reduced ARD in the absence of LepA

Gene	Fold decrease in ARD^a^			
	WT / M	*q*-value^b^	C/M	*q*-value^b^
*yjfN*	128	3.3 × 10^−2^	4	3.0 × 10^−2^
*tdcA*	126	5.0 × 10^−3^	5	4.2 × 10^−2^
*galS*	46	6.1 × 10^−4^	14	4.6 × 10^−3^
*ygeV*	30	4.2 × 10^−3^	5	1.2 × 10^−2^
*lldP*	25	1.3 × 10^−3^	58	4.8 × 10^−3^
*dsdX*	24	5.5 × 10^−3^	97	1.9 × 10^−3^
*ychH*	21	9.3 × 10^−3^	6	6.1 × 10^−3^
*yqeF*	19	4.2 × 10^−3^	5	2.3 × 10^−2^
*cstA*	19	5.3 × 10^−3^	9	1.3 × 10^−3^
*raiA*	18	6.3 × 10^−4^	17	1.9 × 10^−3^
*dctA*	16	6.1 × 10^−4^	11	6.4 × 10^−4^
*cspD*	15	2.6 × 10^−3^	6	7.9 × 10^−3^
*dppA*	15	3.0 × 10^−3^	9	1.2 × 10^−2^
*tnaC*	14	5.3 × 10^−3^	26	3.2 × 10^−3^
*malT*	14	6.1 × 10^−4^	4	1.4 × 10^−3^
*tnaB*	12	5.9 × 10^−3^	90	3.4 × 10^−3^
*aldA*	12	2.0 × 10^−3^	42	6.4 × 10^−4^
*yniA*	11	3.2 × 10^−2^	5	2.4 × 10^−2^
*yejG*	11	1.9 × 10^−2^	2	3.3 × 10^−2^
*fucR*	11	5.2 × 10^−3^	4	2.4 × 10^−2^
*gntP*	11	1.2 × 10^−2^	4	1.7 × 10^−2^
*yeaT*	11	5.8 × 10^−3^	3	1.3 × 10^−2^
*yfiQ*	10	1.0 × 10^−3^	2	6.4 × 10^−3^
*malK*	10	4.0 × 10^−3^	49	3.0 × 10^−4^
*uspF*	10	5.0 × 10^−3^	4	5.5 × 10^−3^
*araC*	10	2.6 × 10^−3^	5	1.7 × 10^−2^
*cdaR*	10	3.6 × 10^−3^	4	1.2 × 10^−2^

^a^ARD was calculated as the percentage of ribosome-footprint reads divided by the percentage of total-mRNA reads for each gene (protein-coding region) of the genome. Shown are quotients representing fold decrease in ARD due to loss of LepA. WT/M, wild-type versus mutant *ΔlepA* strain; C/M, complemented *ΔlepA*(pLEPA) versus mutant *ΔlepA* strain.

^b^Statistical significance was assessed using the Benjamini–Hochberg corrected Student's *t*-test method, comparing differences in log_2_(ARD) values. In all cases shown, *q* < 0.05, indicating statistical significance at the >95% confidence level.

**Figure 3.**
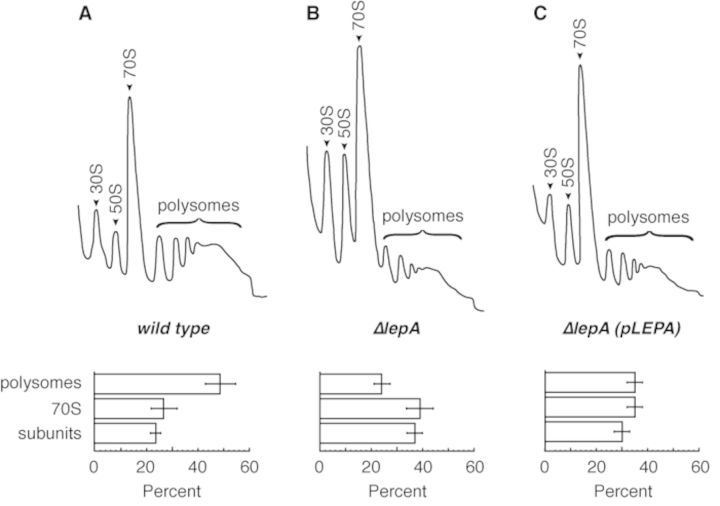
Loss of LepA results in fewer polysomes and more free subunits. Representative *A*_254_ traces of sucrose gradient profiles of wild-type (A), *ΔlepA* (B) and *ΔlepA*(pLEPA) (C) strains, with peaks corresponding to 30S and 50S subunits, 70S monosomes and polysomes (as indicated). The percentage of polysomes, 70S monosomes, and subunits (30S plus 50S) are graphed below (mean ± SEM). Differences deemed significant are seen for polysome levels (WT versus M, *P* = 2.4 × 10^−2^; C versus M, *P* = 1.9 × 10^−2^) and subunit levels (WT versus M, *P* = 2.6 × 10^−2^), based on Student's *t*-test.

In preparing lysates for the ribosome profiling analysis, it was evident that the mutant strain had significantly more free subunits and fewer polysomes than the WT strain (Figure 3). This defect was largely rescued in the complemented *ΔlepA*(pLEPA) strain. These data suggest that ARD is generally reduced in the mutant, in line with the ribosome profiling data.

The observation that many mRNAs were overexpressed in the mutant and yet the corresponding proteins underproduced prompted us to compare the total amount of mRNA in the various strains. Our protocol for construction of the cDNA libraries of total RNA did not include a step to remove rRNA (commonly employed in other studies), and hence we reasoned that read counts should provide a proxy for the relative abundance of various classes of RNA molecules in the cell. Mutation *ΔlepA* increased the fraction of coding mRNA by a factor of 1.2, an effect reversed in presence of pLEPA (Table 3). A proportionally much smaller change was seen for rRNA, indicating that the concentration of mRNA relative to ribosomes increases in the mutant. These data provide further evidence for generally lower ribosome densities in the mutant, and suggest that the actual ARD values in this strain are across-the-board smaller (by ∼16%) than reported in Supplementary Table S3. The collective level of stable small RNAs also increased upon loss of LepA, although the magnitude of this effect is slight (Table 3). The overall level of tRNA was reduced in the mutant strain by 15%, although this effect was not complemented and hence cannot be attributed to the loss of LepA.

**Table 3.** Relative abundance of various types of RNA in cells containing and lacking LepA

Strain	mRNA^a^	tRNA	rRNA	sRNA^b^
Wild-type	4.43 ± 0.02**	14.13 ± 0.05*	65.27 ± 0.10**	5.68 ± 0.02**
*ΔlepA*	5.32 ± 0.10	11.97 ± 0.41	67.60 ± 0.45	5.87 ± 0.04
*ΔlepA* (pLEPA)	3.64 ± 0.39*	11.28 ± 0.79	70.99 ± 1.40*	5.53 ± 0.02**

Data represent percentages of all genome-aligned reads, mean ± SD (*n* = 3). Asterisks denote significant differences from the *ΔlepA* case (**P ≤ 0.01; *P ≤ 0.05), based on Student's *t*-test.

^a^Protein coding regions.

^b^Includes 6S RNA, RNase P RNA, 4.5S RNA, tmRNA and annotated regulatory small RNAs.

### The effect of LepA on ARD is related to the sequence of the TIR

**Figure 4.**
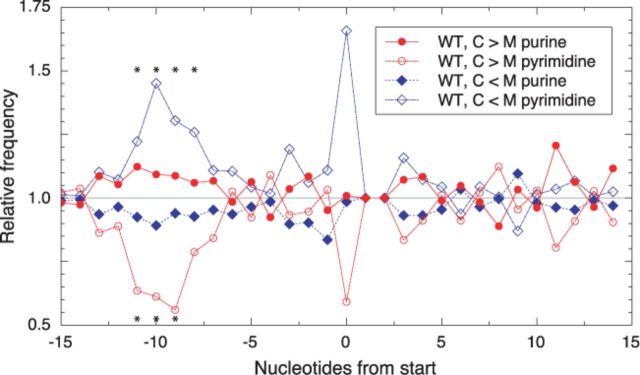
Effects of LepA on ARD are related to the TIR sequence. Nt frequencies at each position of the TIR were determined for the subset of genes with decreased ARD in the absence of LepA (WT, C > M; 237 genes), the subset of genes with increased ARD in absence of LepA (WT, C < M; 283 genes) and all genes analyzed (1870). Purine and pyrimidine frequencies for each subset, relative to those of the complete set, are plotted as a function of TIR position (as indicated; position zero corresponds to the first nt of the start codon). Binomial tests indicate that pyrimidines are significantly underrepresented in the former subset (WT, C > M) at positions −11 (*P* = 7.4 × 10^−4^), −10 (*P* = 2.9 × 10^−3^) and −9 (*P* = 1.6 × 10^−3^) and significantly overrepresented in the latter subset (WT, C < M) at positions −11 (*P* = 3.3 × 10^−2^), −10 (*P* = 3.7 × 10^−4^), −9 (*P* = 2.5 × 10^−2^) and −8 (*P* = 2.6 × 10^−2^), data points marked with asterisks. The differences seen at the first position of the start codon (position zero) are deemed less than statistically significant (only 40 of the 1870 analyzed genes begin with a pyrimidine). Genes *infC* and *pcnB* have the rare start codon AUU and hence were omitted from the analysis. The second and third nts of the start codon (UG) are otherwise invariant and assigned the value of 1.0.

In theory, reduced ARD could result from slower initiation or faster elongation. However, the large effects on ARD observed here (Table 2) are difficult to rationalize as elongation defects. For example, the idea that certain genes are translated with a >10-fold higher overall elongation rate in the mutant seems implausible, particularly in light of evidence that elongation rates are generally uniform across all genes (50,51). Hence, the most straightforward explanation is that, for a number of coding regions, LepA influences the rate of translation initiation substantially. If this is indeed the case, we reasoned that there might exist a relationship between the effect of LepA on ARD and the sequence and/or structure of the TIR. To explore this, we aligned the TIR sequences with respect to the start codon and looked for differences in nt frequencies for those genes with altered ARD in the mutant. Intriguingly, pyrimidines are significantly underrepresented in the SD region of genes for which ARD depends on LepA (WT, C > M) (Figure 4). And the opposite trend is seen for the genes that exhibit increased ARD in the absence of LepA (WT, C < M). Changes in pyrimidine frequency are the more sensitive indicator in this analysis because the SD sequences are characteristically purine rich. These data suggest that initiation in the absence of LepA is more problematic for mRNAs with stronger SD sequences and less problematic for mRNAs with weaker SD sequences. The former acquire a competitive disadvantage in the mutant (lower ARD), while the latter gain a competitive advantage (higher ARD). Importantly, this relationship between TIR sequence and ARD provides additional evidence that LepA contributes to translation initiation, either directly or indirectly.

### Effects of *ΔlepA* on the distribution of ribosomes along mRNAs

**Figure 5.**
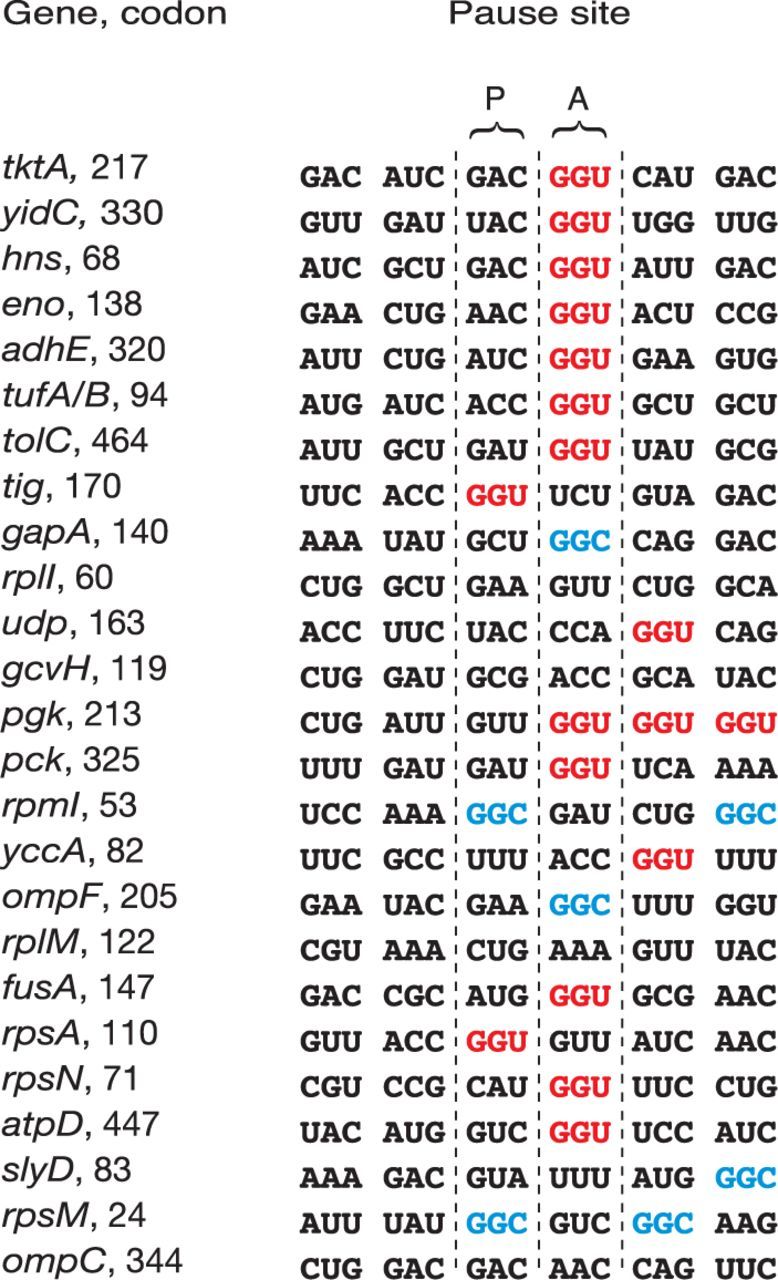
LepA prevents ribosomal pausing at certain GGU codons. Aligned are the coding sequences corresponding to the predicted paused ribosomes seen specifically in the mutant strain. The left column identifies the gene and the codon number (of the P codon of the paused complex). Codon GGU (red) is significantly overrepresented as the A codon (*P* = 7.4 × 10^−13^), based on a two-tailed binomial test. GGC (blue), the other codon recognized by Gly-tRNA^Gly3^, is seen to occupy the A site in two cases, which is deemed less-than-significant enrichment.

Ribosome occupancy tends to vary locally on a given mRNA, due, for example, to ribosome pausing (31). To investigate whether LepA influences elongation, we first performed a metagene analysis to look for general effects of *ΔlepA* on ribosome distribution along mRNA. Plots representing the average ribosome distribution for the 1872 coding regions, aligned with respect to the start codon, revealed lower ribosome occupancy for the first ∼20 codons in the presence of *ΔlepA::cat* (Supplementary Figure S8). However, this trend was just as evident in the complemented strain and hence cannot be attributed to loss of LepA. Next, we looked for complementable changes in the pattern of ribosome occupancy on a gene-by-gene basis, initially by visually scanning the profiling data on the genome browser. Few differences were seen, the most notable being putative pause sites in *ftsW* and *dppA*. While these effects seemed by eye to be complementable, subsequent analysis for each of the corresponding 10 nt windows failed to confirm a significant difference in the mutant versus complemented strains. Finally, we computationally searched for 10 nt windows that exhibited significant differences in local ribosome occupancy (i.e. after normalization with respect to total footprint reads per gene). In the WT versus M comparison, 3185 windows exhibited significant differences, whereas in the C versus M comparison this number was much smaller—187. These data are in line with the metagene analysis, which revealed obvious but non-complementable effects of *ΔlepA::cat* at the 5′ ends of genes (Supplementary Figure S8). Forty-three windows showed significantly higher ribosome occupancy in the mutant strain compared to either strain containing LepA, whereas 13 windows showed lower occupancy. Upon combining consecutive windows, the former 43 windows were reduced to 36 regions, in which we identified 25 clear pauses that are highly position specific (i.e. generally involve a single ribosome footprint) and clearly due to loss of LepA. Notably, RD was not reduced after these pause sites, suggesting that pausing in these cases does not decrease the overall rate of protein synthesis. When the corresponding coding sequences were aligned with respect to the pause position, a glycine codon (GGU or GGC) was often seen to occupy the A site of the paused ribosome (Figure 5). Or, GGU/C was seen one codon away, raising the possibility that A codon assignment may be slightly off in these cases (due to complex-dependent variability of footprint length). A two-tailed binomial test revealed that the occurrence of GGU at the assigned A site is much higher than expected by chance (*P* = 7.4 × 10^−13^), although the same cannot be said of GGC. These data provide evidence that LepA prevents or reduces ribosomal pausing at certain GGU codons in the cell.

Because GGU and GGC codons are only read by Gly-tRNA^Gly3^ in *E. coli* (52), we used acid gel electrophoresis and northern blot analysis (23) to compare Gly-tRNA^Gly3^ levels among the three strains. Both the amount of tRNA^Gly3^ and its charging level were as high in the mutant strain as in the control strains (Supplementary Figure S9). Thus, the observed ribosomal pausing at GGU codons in the absence of LepA is not an indirect consequence of lower Gly-tRNA^Gly3^ concentration.

### LepA does not generally influence polypeptide chain elongation rate

We directly measured elongation rates for the synthesis of LacZ and another large (∼80 kDa) well-resolved protein, in the presence and absence of LepA. A pulse-chase method was employed (53), albeit without dual radiolabeling to correct for handling error. The rates with which full-length proteins accumulate were essentially indistinguishable in WT and *ΔlepA* strains (Supplementary Figure S10). These data indicate that LepA does not generally impact average elongation rate.

## DISCUSSION

### Loss of LepA mainly impacts translation initiation

A number of observations from the Weissman group suggest that ARD is largely determined by the rate of initiation. For example, (i) use of the specific inhibitors harringtonine and cycloheximide allowed elongation rates to be measured globally in eukaryal cells, and the results show remarkable uniformity of elongation rates among genes, regardless of functional class or codon usage (50); (ii) RD for the 5′ and 3′ portions of bacterial genes were found be virtually identical (31,51), providing no evidence that ribosome pausing or stalling normally impacts the overall rate of protein synthesis and (iii) ribosome footprint coverage (per gene length) was found to be proportional to protein synthesis rates in bacterial and eukaryal cells (51), which would only occur if average elongation rates across genes are uniform. In this current study, we show that, in unstressed *E. coli* cells, loss of LepA leads to altered ARD for more than 500 coding regions. In many cases the magnitude of this effect is substantial, with changes in ARD of ≥5-fold for dozens of genes. We also find evidence that LepA influences translation elongation, preventing ribosomal pauses at certain GGU codons. However, these latter effects are relatively few, highly localized (i.e. no impact on RD upstream or downstream), and occur in genes that differ from those that exhibit pronounced LepA-dependent ARD changes. Moreover, LepA does not generally impact average elongation rate, based on direct measurements of synthesis rates for two large polypeptides *in vivo*. Collectively, these observations lead us to conclude that LepA primarily influences initiation, either directly or indirectly. Consistent with this interpretation, we detect a relationship between the TIR sequence and the effect of LepA on ARD. For genes with significantly lower ARD in the mutant (i.e. LepA-dependent), pyrimidines are underrepresented in the SD region; the opposite trend is seen for genes with higher ARD in the absence of LepA.

While the effect of LepA on translation depends on the particular gene, LepA clearly enhances the translation efficiency (i.e. increases ARD) of many genes. In the mutant strain, genes with ≥10-fold lower ARD outnumber those with ≥10-fold higher ARD by 26 to 1, total polysome levels are reduced by half and the overall amount of mRNA per ribosome in the cell is increased by ∼16%. The latter observation implies that the actual ARD values for the mutant are across-the-board smaller than reported and hence the number of LepA-dependent genes larger. Together, these findings suggest that LepA generally promotes translation initiation in the cell.

How might LepA impact initiation? A growing body of evidence indicates that, in all cells, ribosome biogenesis is monitored through quality control mechanisms. In eukarya, for example, late-stage maturation of the 40S subunit involves a ‘test-drive’—a translation-like cycle of initiation-factor-dependent 80S formation followed by release-factor-dependent subunit splitting (54,55). Similar mechanisms exist in bacteria, with later steps occurring in the context of the 70S ribosome (56,57), and immature subunits that escape such quality control checkpoints have clear defects in initiation (58). We propose that LepA plays a role in late-stage ribosome biogenesis and subunits formed in its absence are functionally compromised, particularly with respect to initiation at certain TIRs (Table 2). In line with this hypothesis, one of the genes identified in our synthetic lethal/sick screen is *rsgA* (Table 1), which encodes a GTPase involved in late-stage ribosome maturation (59). RsgA (also called YjeQ) has been shown to catalyze the release of RbfA from the 30S subunit after 17S-to-16S rRNA processing (38). LepA may play a similar and partially redundant role in catalyzing late-stage assembly events, explaining the synthetic phenotype we see. Loss of LepA increases the proportion of free subunits in the cell and confers sensitivity to low temperature (Figure 3, (8)), as is seen for cells lacking any of several known assembly factors (56). Moreover, a number of known assembly factors are GTPases (60,61), and Efl1 (an EF-G/EF-2 paralog like LepA) catalyzes release of Tif6 during 60S subunit maturation in yeast (62,63), providing ample precedent for this hypothesis. Of course, another possibility is that LepA participates directly in the initiation process. LepA might, for example, catalyze a conformational change of the ribosome that effectively increases the dynamics of Shine–Dalgarno–anti-Shine–Dalgarno (SD-ASD) interactions, thereby facilitating TIR engagement and/or TIR clearance. Further experiments will be needed to test these possibilities and elucidate the mechanism by which LepA influences translation initiation.

### Effects of LepA on elongation are codon-specific and comparatively subtle

A widely held view is that LepA directly modulates translation elongation (64). This is largely based on the structural similarity between LepA and EF-G and the ability of LepA to affect elongation *in vitro*. Our profiling data suggest that LepA prevents pausing at certain GGU codons, generally consistent with a role for LepA in translation elongation. However, it is important to keep in mind that these apparent effects on elongation are codon-specific and comparatively subtle. For example, the pauses observed in the mutant strain do not notably affect ribosome traffic upstream or downstream, suggesting that the overall rate of protein synthesis in these cases is unchanged. By contrast, the effects of LepA on ARD are numerous and in many cases large, indicative of widespread and substantial changes in gene expression. Hence, we propose that LepA primarily contributes to initiation and secondarily contributes to elongation. A minor secondary role for LepA in elongation is consistent with other *in vivo* data: (i) LepA has no effect on the fidelity of decoding or the frequency of frameshifting in various contexts and conditions (22), (ii) LepA does not generally influence average elongation rate (Supplementary Figure S10) and (iii) LepA fails to inhibit tmRNA-dependent peptide tagging or A-codon cleavage, unless the factor is overexpressed (22).

The basis of the observed ribosome pausing at GGU codons remains unclear, as does the mechanism by which LepA prevents such pauses. Levels of Gly-tRNA^Gly3^ are as high in the mutant strain as in either control strain (Supplementary Figure S9), arguing against the idea that pausing is a consequence of ‘hungry’ ribosomes awaiting cognate substrate. Although both GGU and GGC are recognized by tRNA^Gly3^, only GGU appears significantly overrepresented in the A site of the paused complexes. This implies that the pausing is codon-specific and entails either slower decoding of GGU or slower translocation of GGU-paired peptidyl-tRNA^Gly3^. Whether LepA acts directly or indirectly to promote one of these events remains an open question.

While LepA might act directly to speed elongation at certain GGU codons, our biochemical experiments lend no support to the idea that LepA acts by catalyzing reverse translocation (10). We have been unable to reproduce the toeprinting results of Qin *et al.*, despite an extensive effort to do so (Supplementary Figures S3–S6). In our hands, LepA exhibits ribosome-dependent GTPase activity but fails to promote reverse translocation. Consistent with our results, Cooperman *et al.* have shown that addition of LepA to POST-state ribosomes induces tRNA movement within the 50S subunit but fails to accelerate codon–anti-codon movement within the 30S subunit (12). These data call into question the claim that LepA can catalyze reverse translocation (10).

### Altered gene expression likely explains most synthetic phenotypes of *ΔlepA*

Many of the genes identified as interaction partners of *lepA* contribute to respiration and/or transport (Table 1). Why strains compromised for these cellular functions are particularly sensitive to the absence of LepA is not obvious. We suspect that this is an indirect consequence of altered gene expression due to loss of LepA. Consistent with this idea, *dksA* was among the genes identified in the synthetic lethal/sick screen. The DksA protein binds RNA polymerase and, together with the alarmone (p)ppGpp, regulates transcription of many genes (37). We envisage that, in the context of the single *ΔlepA* mutant, transcriptional regulatory networks largely compensate for altered translation in the cell (and hence no obvious growth defect is seen). However, in the absence of DksA, the transcriptional response is compromised, unmasking a *ΔlepA* phenotype. Notably, characteristic of growth defects previously attributed to *ΔlepA* cells is a lengthened lag phase (8,22,65). Such trouble in growth transitions is exactly what one would expect for cells in which translation initiation is compromised and the normal gene expression network perturbed.

### Non-complementable effects of *ΔlepA::cat*

Mutation *ΔlepA::cat* causes reduced RD at the 5′ end of coding regions (Supplementary Figure S8). The origin of this effect remains unclear. Plasmid-encoded LepA fails to (even partially) reverse this effect, and hence it cannot be attributed to the loss of LepA. The mutation entails replacement of the *lepA* gene with a DNA cassette carrying the *cat* gene and its upstream promoter element, oriented in the same direction as the *lep* operon. Insertion of the *cat* cassette has polar effects on the downstream *lepB* gene (encoding leader peptidase), which might be responsible (via an unclear mechanism) for the ribosome distribution changes seen (Supplementary Figure S8). The *lepB* mRNA level is increased by about 4-fold in the M and C strains, based on read coverage of the 3′ half of gene (pLEPA contains the 5′ part of *lepB* (22), complicating analysis of that region). Although the mRNA level is increased, the level of translation (based on ribosome footprint coverage) is modestly reduced in both the M and C stains (by 40–50%), perhaps because the *cat* cassette disrupts translational coupling that normally contributes to *lepB* translation. Aside from a polar effect on *lepB*, another possibility is that Cm acetyltransferase is somehow responsible for the observed changes in ribosome distribution, as the *cat* gene is uniquely absent from the WT strain.

### Concluding remarks

A major finding of this work is that LepA contributes predominantly to the initiation phase of protein synthesis in *E. coli*. Whether LepA acts directly or indirectly to facilitate TIR selection remains to be determined. For two reasons we favor the hypothesis that LepA acts during ribosome assembly/maturation and hence indirectly contributes to initiation. First, *ΔlepA* confers a synthetic phenotype in the absence of RsgA, a GTPase known to catalyze release of RbfA during late-stage ribosome maturation (38,59). An analogous (and partially redundant) role for LepA would explain the genetic interaction we see. Second, LepA is uniquely conserved in bacteria and bacterial-derived organelles. These lineages have in common the problem of assembling structurally related ribosomes but they exhibit disparate mechanisms of TIR selection. Initiation in many bacteria (e.g. Bacteroidetes, certain Cyanobacteria) and mitochondria, for example, does not entail SD-ASD interactions (66–68). We envisage that LepA participates in a conserved aspect of bacterial ribosome assembly, and subunits formed in its absence exhibit defects with variable consequences depending on the particular organism/organelle (69,70).

## SUPPLEMENTARY DATA

Supplementary Data are available at NAR Online.

SUPPLEMENTARY DATA
