# Supplementary material for: The conserved GTPase LepA contributes mainly to translation initiation in Escherichia coli
Source: Nucleic Acids Res. 2014 Nov 6;42(21):13370–83. doi: 10.1093/nar/gku1098 (PMC4245954; doi:10.1093/nar/gku1098)
Supplement: SUPPLEMENTARY DATA [file supp_42_21_13370__index.html]

The conserved GTPase LepA contributes mainly to translation initiation in Escherichia coli — The conserved GTPase LepA contributes mainly to translation initiation in Escherichia coli — SUPPLEMENTARY DATA 

# The conserved GTPase LepA contributes mainly to translation initiation in *Escherichia coli*

## SUPPLEMENTARY DATA

**Files in this Data Supplement:**

- SUPPLEMENTARY DATA
- SUPPLEMENTARY DATA
- SUPPLEMENTARY DATA
- SUPPLEMENTARY DATA
