# Supplementary material for: The conserved GTPase LepA contributes mainly to translation initiation in Escherichia coli
Source: Nucleic Acids Res. 2014 Nov 6;42(21):13370–83. doi: 10.1093/nar/gku1098 (PMC4245954; doi:10.1093/nar/gku1098)
Supplement: SUPPLEMENTARY DATA [file supp_gku1098_nar-02711-v-2014-File010.pdf]

Supplementary Figures

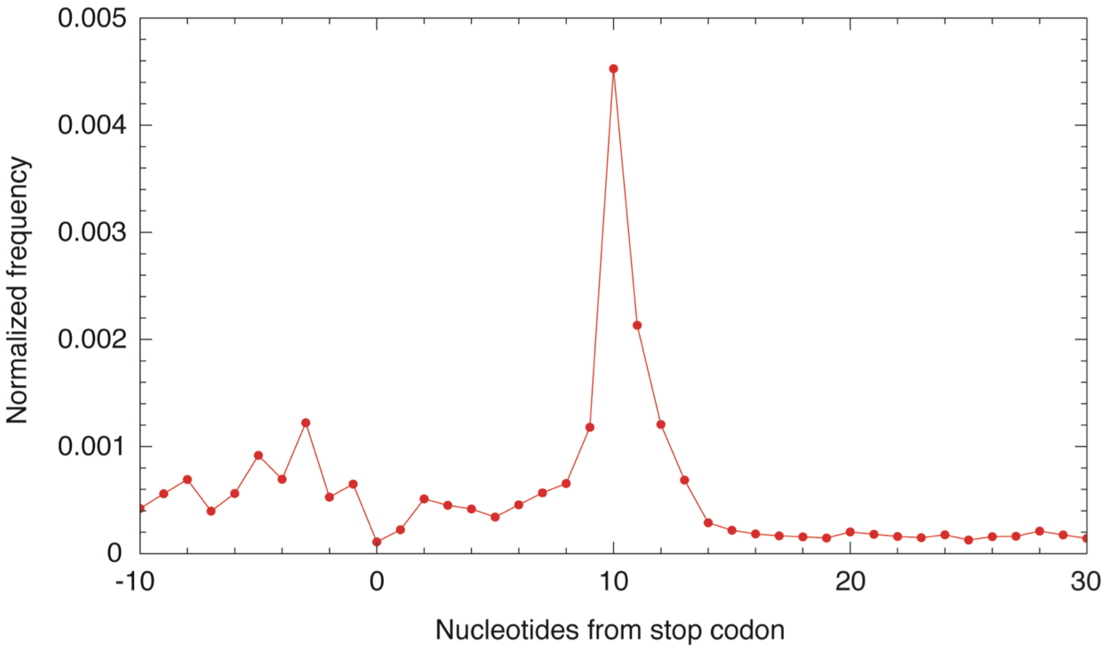

Figure S1. Ribosome footprint fragments end 14 nts downstream of the center of the P site. The coverages of 3’ end positions of ribosome footprint fragments were normalized for each gene to the total RNA coverage and length of the genes, indexed by their distances from the stop codons of their respective genes, and averaged over all genes, all three conditions, and all replicates (0 representing the third codon position of the stop codon). The peak at 10 nts beyond the stop codon, which we interpret as stemming from ribosomes located with the stop codon in their A site, indicates that the center of the P site is 14 nts upstream of the most prevalent 3’ end of the ribosome footprint fragments.

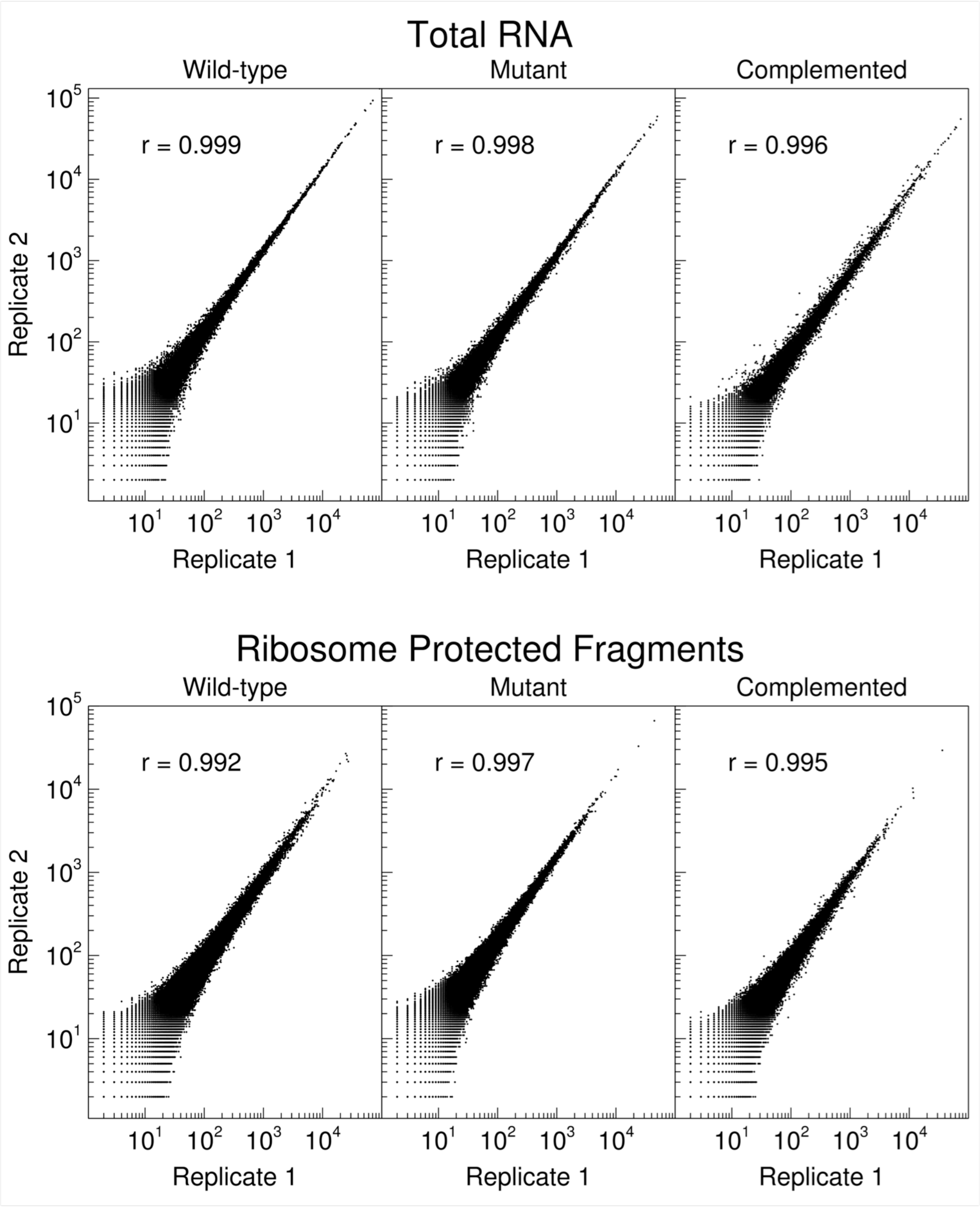

Figure S2. Total RNA and ribosome footprint coverage is highly replicable. Shown are biological replicate 1 and 2 compared to each other in each of the strains (wild-type, mutant, complemented) for the two library types (total RNA and ribosome footprints), comparing the raw reads coverage for each genomic location. The Pearson correlation coefficients for all replicate-to-replicate comparisons are as follows: WT total RNA, r = 0.999, 0.997, and 0.998; M total RNA, r = 0.998, 0.997, and 0.998; C total RNA, r = 0.996, 0.962, and 0.968; WT ribosome footprints, r = 0.992, 0.990, and 0.995; M ribosome footprints, 0.997, 0.976, and 0.981; C ribosome footprints, r = 0.995, 0.997, and 0.995.

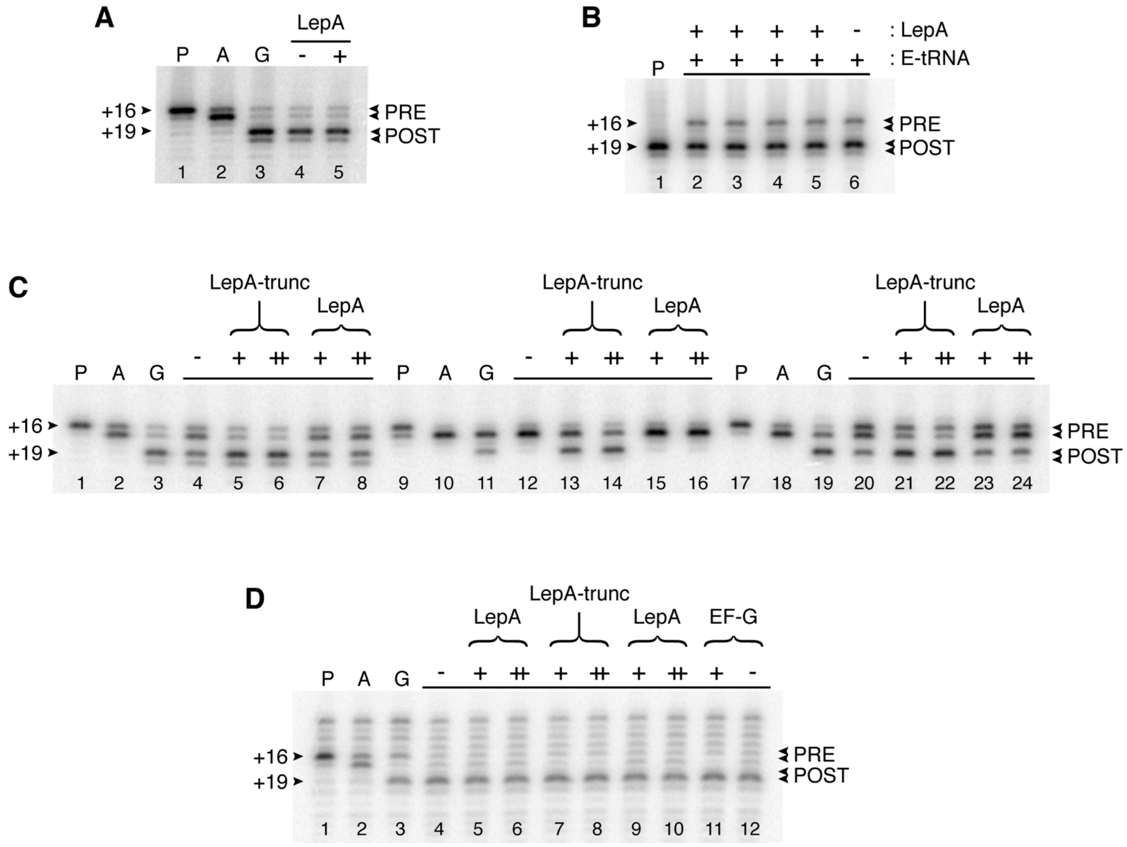

Figure S3. All independent preparations of LepA lack reverse translocase activity. Examples of toeprinting experiments to test the effect of LepA on codon-anticodon movement in the ribosome are shown. Aliquots were removed at various stages of the experiment for primer extension analysis, as described (Shoji et al, 2006). Position +1 is defined as the first nucleotide of the AUG start codon for each of the mRNAs used. (A) Pretranslocation (PRE) complexes were formed by incubating ribosomes (1 μM) in polymix buffer [5 mM potassium phosphate (pH 7.3), 95 mM KCl, 5 mM $Mg(OAc)_2$, 0.5 mM $CaCl_2$, 5 mM NH_4Cl, 8 mM putriscine, 1 mM spermidine, 1 mM DTT] with m291 [1 μM, (Shoji et al, 2006)] and $tRNA^{fMet}$ (1 μM) for 20 min at 37°C to bind the P site (P lane) and then adding *N*-acetyl-Phe-$tRNA^{Phe}$ and further incubating for 10 min to fill the A site (A lane). Next, His6-EF-G (1 μM) and GTP (300 μM) were added to form the posttranslocation (POST) complex (G lane), and the reaction was then depleted of EF-G using Ni-NTA resin (Shoji et al, 2006) and further incubated for 5 min with GTP (300 μM) and with or without LepA prep 1 (2 μM) as indicated. (B) Ribosomes (1 μM) were incubated in Nierhaus buffer (Qin et al, 2006) [20 mM HEPES-KOH (pH 7.5), 4.5 mM $Mg(OAc)_2$, 150 mM NH_4OAc, 2 mM spermidine, 0.05 mM spermine, 4 mM BME] with message m291 (0.5 μM) and *N*-acetyl-Phe-$tRNA^{Phe}$ (1 μM) at 37°C to bind the P site (P lane). Then, $tRNA^{fMet}$ (1 μM) was added to bind the E site, with GTP (1 mM) and with or without LepA (0.7 μM) as indicated, and the reactions were incubated for 5 min prior to toeprint analysis. For lanes 2, 3, 4, and 5, LepA preparations 5, 6, 7 and 8 were used, respectively. Note that the degree of reverse translocation seen after 5 min [one-fifth the mean time for this reaction (Shoji et al, 2006)] is the same in the presence and absence of LepA. (C) LepA preparations 1 and 2 were tested for reverse translocase activity using ribosome complexes programmed with m291 (lanes 1-8), m292 [(Shoji et al, 2006), lanes 9-16], and m293 [(Shoji et al, 2006), lanes 17-24]. PRE complexes were formed by incubating programmed ribosomes (1 μM) in Nierhaus buffer with $tRNA^{fMet}$ (2 μM) for 20 min at 37°C to bind the P site (P lanes; 1, 9, 17) and then adding the appropriate *N*-acetyl-aminoacyl-tRNA (2 μM) to

bind the A site (A lanes; 2, 10, 18). EF-G (0.3 μM) and GTP (120 μM) were added next, forming the POST complex (G lanes; 3, 11, 19). Each complex was then pelleted through a 2 mL 10% sucrose cushion in Nierhaus buffer (65,000 g, 18 h) to remove EF-G (Qin et al, 2006). Pellets were dissolved in Nierhaus buffer, and the ribosome complexes were further incubated for 10 min at 37°C with GTP (100 μM) and without or with LepA (prep 1; 1 or 5 μM) or LepA-trunc (prep 2; 1 or 5 μM) as indicated, prior to primer extension. Note that spontaneous reverse translocation is observed in all complexes upon EF-G removal (lanes 4, 12, 20), but neither preparation of LepA promotes this reaction. Rather, LepA-trunc uniquely promotes forward translocation (lanes 5-6, 13-14, 21-22). (D) Testing for reverse translocase activity under conditions virtually identical to those described previously (Qin et al, 2006). PRE complexes were formed by incubating ribosomes (1 μM) in Nierhaus buffer with MF-mRNA (20 nM) and $tRNA^{fMet}$ (2 μM) to bind the P site (P lane), and then adding *N*-acetyl-Phe-$tRNA^{Phe}$ (2 μM) to bind the A site (A lane). Next, EF-G (0.3 μM) and GTP (120 μM) were added to form the POST complex (G lane), which was then pelleted through a sucrose cushion as described above to remove EF-G (Qin et al, 2006). Finally, the ribosome complexes were dissolved in Nierhaus buffer and further incubated for 10 min with GTP (120 μM) in the absence or presence of LepA prep 3 (lanes 5 and 6, 1 and 5 μM), prep 2 (LepA-trunc; lanes 7 and 8, 1 and 5 μM), prep 1 (lanes 9 and 10, 1 and 5 μM), or EF-G (lane 11, 1 μM), as indicated, prior to primer extension. Note that none of the added factors promotes reverse translocation.

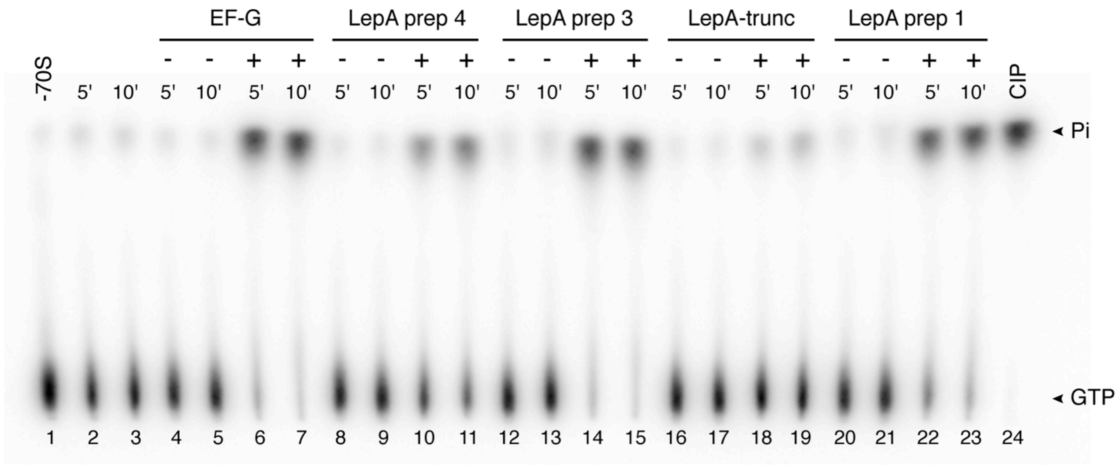

Figure S4. LepA catalyzes GTP hydrolysis in the presence of ribosomes. Radiolabeled [γ-^{32}P]-GTP (50 μM) was incubated at 37°C in polymix buffer [5 mM potassium phosphate (pH 7.3), 95 mM KCl, 5 mM $Mg(OAc)_2$, 0.5 mM $CaCl_2$, 5 mM NH_4Cl, 8 mM putriscine, 1 mM spermidine, 1 mM DTT] for 5 or 10 min (as indicated) with ribosomes (1 μM) and in the absence or presence of EF-G or various preps of LepA (1 μM, as indicated). A negative control reaction without ribosomes or factors (-70S, lane 1) was also included, as was a positive control containing calf intestinal phosphatase (CIP, lane 24) to completely hydrolyze GTP. Aliquots (2 μL) were removed at each time point and mixed with 2 μL quench solution (1 M $HClO_4$, 3 mM KH_2PO_4). Products were resolved by thin layer chromatography on PEI-cellulose as described (Walker et al, 2008). Note that LepA preps 1 and 3 exhibit robust GTPase activity, comparable to that of EF-G, whereas preps 2 (LepA-trunc) and 4 (purified from the insoluble fraction) show lower activity.

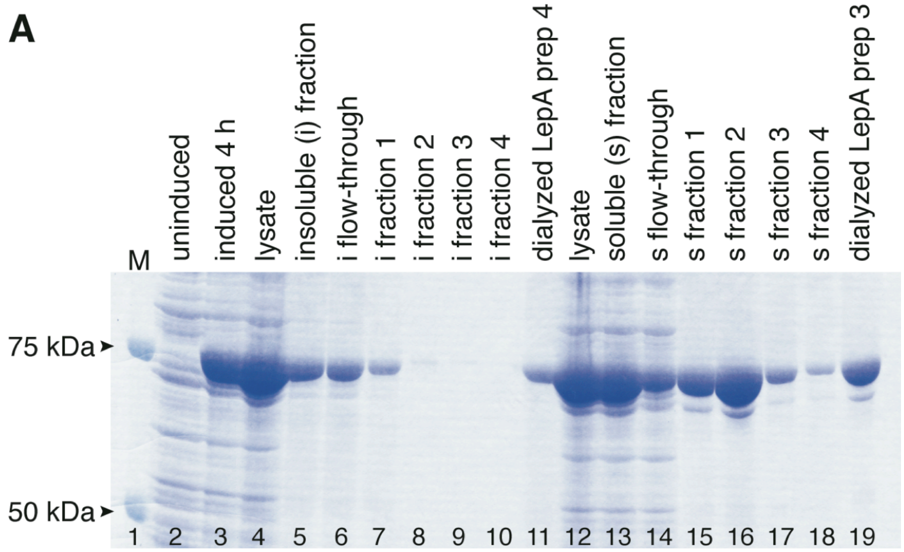

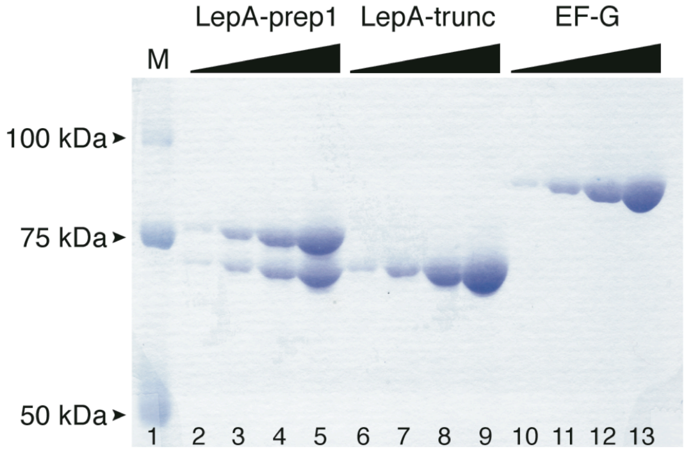

Figure S5. SDS-PAGE analysis of various preparations of LepA. (A) An SDS-PAGE gel stained with Coomassie Blue showing various protein fractions during the purification of LepA, prep 3 (lanes 12-19) and prep 4 (lanes 4-11), as indicated. Lane 1 contains molecular weight markers, and lanes 2 and 3 contain total cellular lysate before and after induction with IPTG. (B) Comparison of purified proteins LepA prep 1 (lanes 2-5), prep 2 (LepA-trunc, lanes 6-9) and EF-G (lanes 10-13). In each case, increasing amounts of protein (20, 50, 100, 200 pmol) were loaded in adjacent lanes.

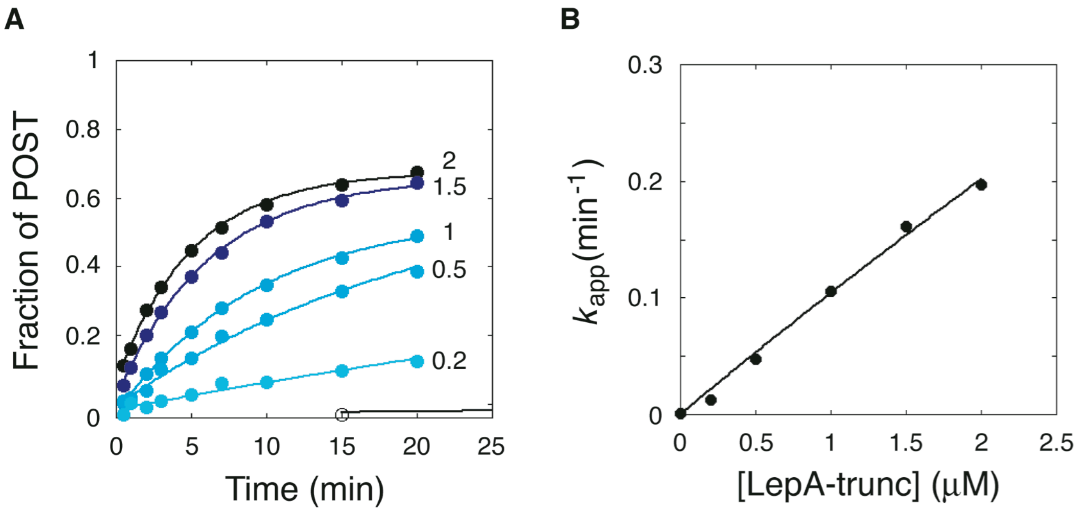

Figure S6. LepA-trunc catalyzes forward translocation. (A) PRE complexes were formed on m291 as described for Supplementary Fig. S3A and diluted to 0.25 μM. LepA-trunc (various micromolar concentrations, as indicated) and GTP (300 μM) were added, and the extent of translocation at various time points was measured by toeprinting. Data were fit to a single-exponential function to determine apparent rates. Essentially no translocation was seen in the absence of added factor (open circles), a control reaction monitored for 90 min, consistent with earlier studies of this complex (Shoji et al, 2006). (B) Apparent rates were plotted as a function of input LepA-trunc concentration, and the data were fit to the equation $k_{app} = k_{cat}$•[LepA-trunc] / (K_M + [LepA-trunc]), yielding the parameters k_{cat} = 3.2 min^{-1} and K_M = 30 μM.

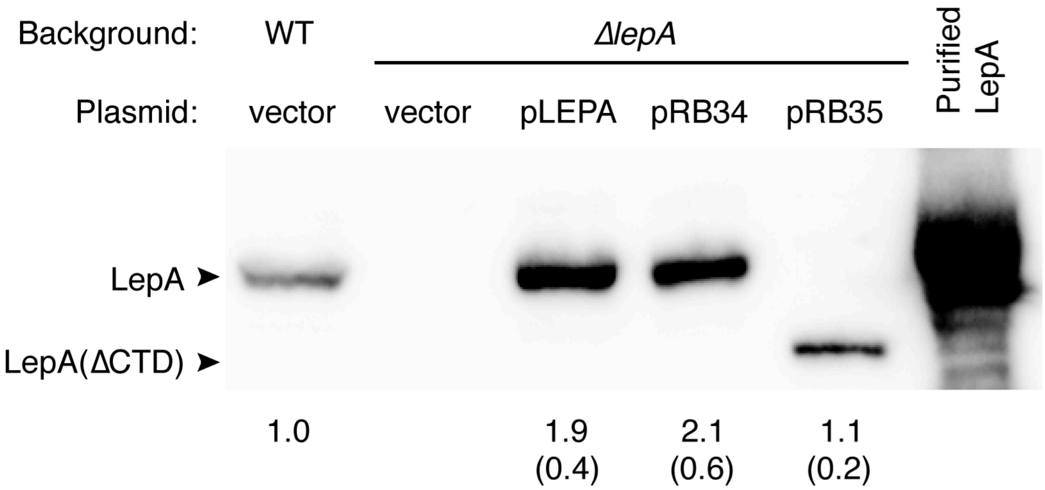

Figure S7. Cellular levels of plasmid-encoded mutant proteins LepA(H81A) and LepA(Δ487-594) are comparable to that of LepA in control strains. Lysates from isogenic wild type (WT) and mutant (Δ*lepA*) strains harboring the vector only, pLEPA, pRB34, or pRB35 were subjected to Western blot analysis. The positions of the full-length protein (LepA) and the Δ487-594 deletion variant [LepA(ΔCTD)] are indicated. The relative amount of protein with respect to LepA in wild type cells is shown below the lanes [mean ± (range); n = 2].

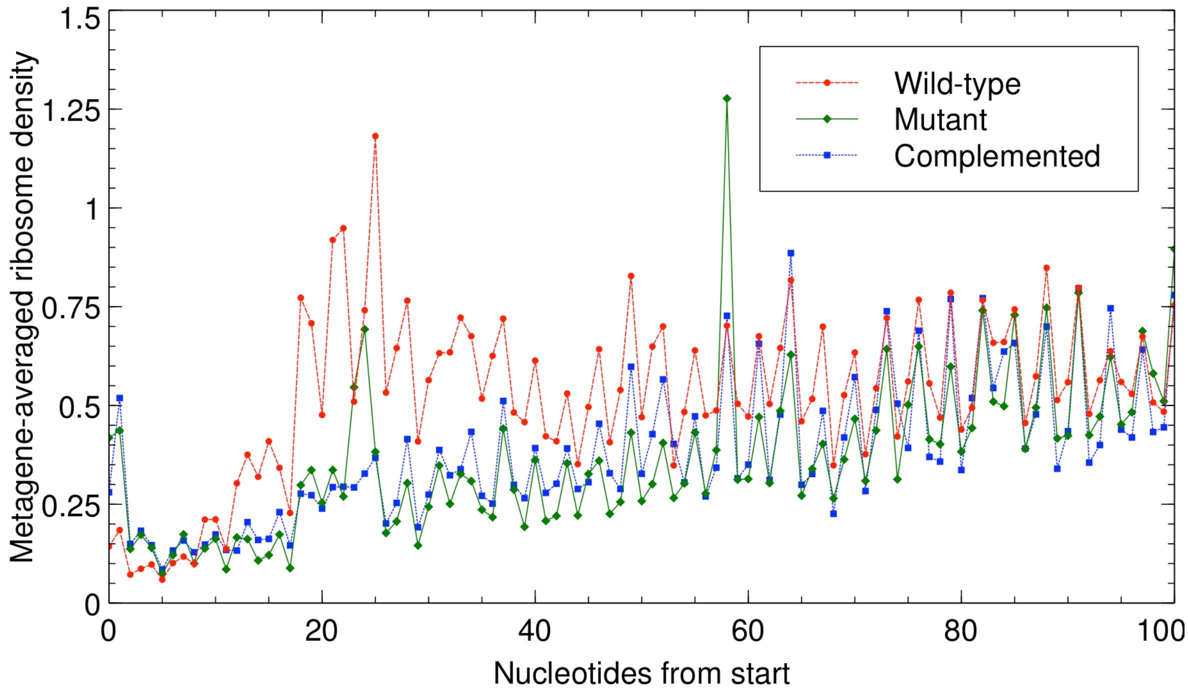

Figure S8. Metagene analysis reveals generally reduced ribosome density at the 5' end of coding regions for both the mutant and complemented strains. Ribosome density values were calculated for each gene position and then averaged across all high-coverage genes. Shown are plots of metagene-averaged ribosome density as a function of gene position (codons 1-33) for the WT, M, and C strains (as indicated).

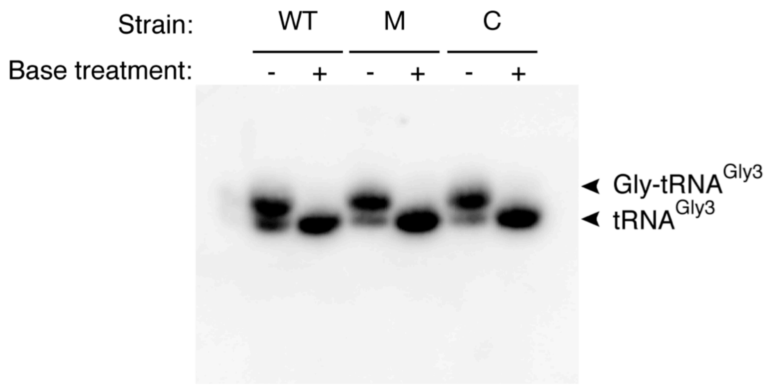

Figure S9. Loss of LepA does not reduce the level of Gly-tRNA^{Gly3} in the cell. Total aminoacyl-tRNA was extracted from WT, M, and C strains under acidic conditions, portions were deacylated with base treatment (as indicated), and samples were subjected to acid gel Northern analysis using a tRNA^{Gly3}-specific, [^{32}P]-labeled, oligonucleotide probe.

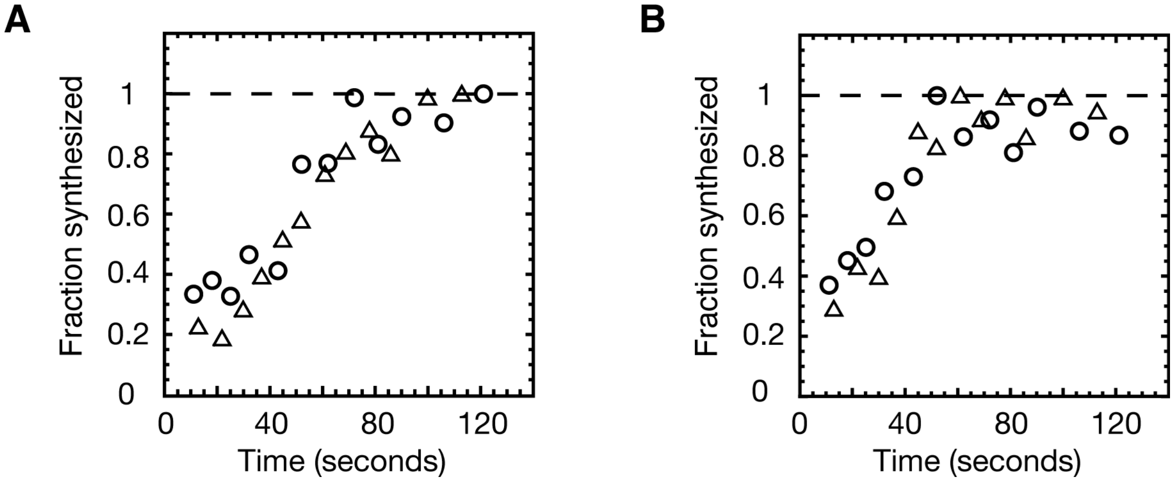

Figure S10. LepA has no appreciable effect on the rate of polypeptide chain elongation measured *in vivo*. To WT and M cells in logarithmic phase, ^{35}S-methionine was added 10 s before an excess of unlabeled methionine. Then, aliquots were removed at various time points, treated with chloramphenicol to stop elongation, and analyzed by SDS-PAGE. The amount of full-length labeled LacZ (A) and another large well-resolved protein (B) are plotted as a function of time, allowing the average rate of elongation to be compared in the WT (○) and M (△) cells. The identity of the latter protein is unknown; it was analyzed because it was well resolved and efficiently labeled in the experiment.

Supplementary Materials and Methods

Purifications of LepA

LepA was purified 11 times, using 3 different overexpression constructs. In the first preparation, LepA with an *N*-terminal hexa-histidine (His6) tag and the *C*-terminal extension GLCGR was overproduced in strain JW2553 harboring the ASKA-*lepA* plasmid (Kitagawa et al, 2005). Cells were grown to mid-logarithmic (mid-log) phase ($OD_{600} \approx 0.5$) in 1 L LB medium supplemented with $MgCl_2$ (100 mM), induced by addition of IPTG (1 mM), and harvested 4 hours later. All subsequent steps were performed at 4°C. Cells were lysed using the French press in 15 mL lysis buffer [50 mM Tris-HCl pH 7.6, 60 mM KCl, 10% glycerol, 6 mM β-mercaptoethanol (BME)]. The lysate was clarified by centrifugation and incubated with 2 mL Ni-NTA resin (Qiagen, equilibrated in lysis buffer) for 1 hour in a capped tube on a slow rotator. The protein-bound resin was packed into a column and washed with 40 mL wash buffer (50 mM Tris-HCl pH 7.6, 300 mM KCl, 10% glycerol, 6 mM BME, 10 mM imidazole), using a low-pressure pump system. The bound protein was eluted with elution buffer (50 mM Tris-HCl pH 7.6, 40 mM KCl, 10% glycerol, 6 mM BME, 500 mM imidazole), dialyzed against storage buffer (50 mM Tris-HCl pH 7.6, 50 mM KCl, 10% glycerol, 6 mM BME), flash frozen in small aliquots, and stored at -70°C. Subsequent SDS-PAGE analysis revealed that this preparation of LepA (prep 1) was a mixture of full-length and C-terminally truncated protein (Fig. S5).

We were concerned that the inability of prep 1 to promote reverse translocation might be due to the *C*-terminal tag (GLCGR), predicted from DNA sequencing of the ASKA construct. Hence, we introduced a stop codon into this plasmid to effectively remove this peptide tag. The resulting plasmid, pSS29, encodes His6-LepA with the native C-terminus. The sequence of the entire *lepA* coding region of pSS29 was confirmed to contain no mutations. In the second preparation of LepA, protein overexpressed from pSS29 in strain C2992 [F′ *proA*$^+$*B*$^+$ *lacI*^{q} *Δ(lacZ)M15 zzf::Tn10* (Tet^R) / *fhuA2Δ(argF-lacZ)U169 phoA glnV44 Φ80Δ(lacZ)M15 gyrA96 recA1 relA1 endA1 thi-1 hsdR17;* from New England Biolabs] was purified using Ni-NTA resin followed by anion exchange (Resource Q, GE Healthcare) chromatography. The initial steps of the procedure were similar to those of prep 1 except that cells were grown in 0.5 L LB without added $MgCl_2$ (true of all subsequent preps), and the concentration of glycerol in the lysis, wash, and elution buffers was reduced to 5%. Protein bound to Ni-NTA was washed in batch 5 times, and the eluate was dialyzed overnight against Q buffer (50 mM Tris-HCl pH 7.3, 20 mM KCl, 5% glycerol, 6 mM BME). The sample was then loaded onto an FPLC Resource Q column (pre-equilibrated with the same buffer), washed with multiple column-volumes of Q buffer, and eluted with a salt gradient (20-500 mM KCl over 10 column volumes). Fractions deemed pure by SDS-PAGE were pooled (prep 2), dialyzed against storage buffer, flash frozen in small aliquots, and stored at -70°C.

Prep 2 was found to be a C-terminally truncated variant of LepA (LepA-trunc; see Results, Supplementary Figs. S3-S6). Thus, we moved pSS29 into strain BL21/DE3 [F^- *ompT gal dcm lon* $hsdS_B(r_B^- \, m_B^-)$ *λDE3 (lacI lacUV5-T7 gene 1 ind1 sam7 nin5)*], and included the protease inhibitor PMSF throughout the procedure. Full-length protein overproduced from this strain was purified from both the soluble (prep 3) and insoluble fractions (prep 4). For prep 3, Ni-NTA affinity chromatography was employed as detailed above for prep 1, except that the lysis, wash, and elution buffers contained 0.1 mM PMSF and 5% glycerol. For prep 4, the insoluble fraction of the lysate was dissolved in extraction buffer (50 mM Tris-HCl pH 7.3, 60 mM KCl, 5% glycerol, 6 mM BME, 0.1 mM PMSF, 7 M urea), and the clarified extract was combined with Ni-NTA resin and incubated for 1 hour with gentle rotation. Bound resin was packed into a column, washed with denaturing wash buffer (50 mM Tris-HCl pH 7.3, 300 mM KCl, 5% glycerol, 10 mM imidazole, 6 mM BME, 0.1 mM PMSF, 7 M urea), and then subjected to a reverse urea gradient (7 to 0 M urea) to facilitate refolding of LepA on the column. Elution and subsequent steps were performed as described for prep 3.

Because none of preps 1-4 showed reverse translocase activity, we requested and received the overexpression construct, pET28a-LepA, from M. Pech and K. Nierhaus, along with their advice on overproduction and purification procedures. Plasmid pET28a-LepA was transformed into

BL21/DE3(pLysE) (Studier, 1991), and 3 fresh colonies were used to inoculate 3 independent vials of 2 mL LB containing kanamycin (30 μg/mL) and glucose (0.4%), and these starter cultures were grown overnight. In the morning, these cultures were used to inoculate 1 L of the same media, and cultures were grown until mid-log phase ($OD_{600} \approx 0.3$), induced with IPTG (1 mM), and harvested 30 min later ($OD_{600} \approx 0.5$). LepA was purified from the soluble fraction of each of the corresponding lysates using Ni-NTA affinity chromatography, followed by gel filtration chromatography (FPLC Superdex 75) and dialysis against storage buffer (defined above). Lysis, wash, and elution buffers contained PMSF (as for prep 3), whereas the Superdex 75 buffer (50 mM Tris-HCl pH 7.3, 1M KCl, 5% glycerol, 6 mM BME) and storage buffer did not. None of the resulting LepA preparations (5-7) had reverse translocase activity.

Aware of the evidence that *lepA* overexpression causes toxicity (Qin et al, 2006), we repeated the purifications once again, choosing those transformants that appeared to grow more slowly (although colony sizes and culture growth rates were quite uniform under the conditions employed). Twenty fresh BL21/DE3 (pLysE, pET28a-LepA) colonies were used to inoculate starter cultures and grown overnight. In the morning, four cultures yet to reach saturation were used to inoculate 1 L of fresh media, and purifications 8-11 were performed as described above for preps 5-7. None of preparations 8-11 exhibited reverse translocase activity.

References

Kitagawa M, Ara T, Arifuzzaman M, Ioka-Nakamichi T, Inamoto E, Toyonaga H, Mori H (2005) Complete set of ORF clones of Escherichia coli ASKA library (a complete set of E. coli K-12 ORF archive): unique resources for biological research. *DNA Research : An International Journal for Rapid Publication of Reports on Genes and Genomes* **12:** 291-299

Qin Y, Polacek N, Vesper O, Staub E, Einfeldt E, Wilson DN, Nierhaus KH (2006) The highly conserved LepA is a ribosomal elongation factor that back-translocates the ribosome. *Cell* **127:** 721-733

Shoji S, Walker SE, Fredrick K (2006) Reverse translocation of tRNA in the ribosome. *Molecular Cell* **24:** 931-942

Studier FW (1991) Use of bacteriophage T7 lysozyme to improve an inducible T7 expression system. *Journal of Molecular Biology* **219:** 37-44

Walker SE, Shoji S, Pan D, Cooperman BS, Fredrick K (2008) Role of hybrid tRNA-binding states in ribosomal translocation. *Proceedings of the National Academy of Sciences of the United States of America* **105:** 9192-9197
